# Withaferin A triggers G2/M arrest and intrinsic apoptosis in glioblastoma cells via ATF4‐ATF3‐CHOP axis

**DOI:** 10.1111/cpr.12706

**Published:** 2019-10-23

**Authors:** Qin Tang, Liwen Ren, Jinyi Liu, Wan Li, Xiangjin Zheng, Jinhua Wang, Guanhua Du

**Affiliations:** ^1^ The State Key Laboratory of Bioactive Substance and Function of Natural Medicines Institute of Materia Medica Chinese Academy of Medical Science and Peking Union Medical College Beijing China; ^2^ Key Laboratory of Drug Target Research and Drug Screen Institute of Materia Medica Chinese Academy of Medical Science and Peking Union Medical College Beijing China

**Keywords:** apoptosis, cell cycle, endoplasmic reticulum, glioblastoma, unfolded protein response

## Abstract

**Objective:**

Withaferin A (WA) is a bioactive compound with a remarkable anti‐cancer effect derived from *Withania somnifera*, commonly known as ashwagandha. However, the anti‐cancer mechanisms of WA in glioblastoma multiforme (GBM) are still unclear.

**Materials and Methods:**

Cell viability assays and xenografted nude mice were used to evaluate the effects of WA, along with flow cytometry to detect apoptosis and cell cycle of GBM. RNA‐seq analysis, Western blotting, immunofluorescence staining, qRT‐PCR and siRNA gene silencing were carried out to determine the signalling pathways affected by WA.

**Results:**

Withaferin A significantly inhibited the growth of GBM in vitro and in vivo and triggered the intrinsic apoptosis of GBM cells by up‐regulating expression of Bim and Bad. WA arrested GBM cells at the G2/M phase of the cell cycle through dephosphorylating Thr^161^ of CDK1 by activating p53‐independent p21 up‐regulation. Knockdown of p21 restored cell cycle progression and cell viability by down‐regulating the expression of Bad rather than Bim. We demonstrated that endoplasmic reticulum (ER) stress induced by WA through the ATF4‐ATF3‐CHOP axis, initiated apoptosis and G2/M arrest in GBM cells.

**Conclusion:**

We revealed a novel pathway that elucidated WA activation of apoptosis and G2/M arrest in GBM cells through the ATF4‐ATF3‐CHOP axis. This discovery is important for optimization of WA‐based regimens for prevention and/or treatment of GBM.

## 1 INTRODUCTION

Glioblastoma multiforme (GBM, WHO grade IV astrocytoma) is the most biologically aggressive, frequent and lethal subtype of malignant gliomas originating in the central nervous system.^1^ The current standard therapy for GBM patients is surgical resection followed by adjuvant radiation therapy and chemotherapy with oral temozolomide. However, the therapy only improves the median survival from 12.1 to 14.6 months and increases the percent of patients alive at 2 years from 10% to 26%,^2^ while >90% of GBM tumours recur at the original site.^3^ Updated survival data in the United States revealed that 10% of patients receiving the combined treatment were alive at 5 years vs 2% with radiation alone.^4^ Therefore, there is an urgent clinical need for the development of new drugs as GBM therapeutics.

Withaferin A is a bioactive compound derived from the ashwagandha plant, *Withania somnifera*, which has been used for safe and effective treatment of various ailments in Indian Ayurvedic medicine for centuries. Pharmacological studies of the effects of WA have shown that it is anti‐diabetic,^5^ protects liver from acetaminophen‐induced injury,^6^ prevents mammary cancer,^7^ inhibits tumour cell invasion and metastasis,^8^, ^9^ induces cell cycle arrest 10, 11 and triggers apoptosis in breast, prostate, colorectal, non‐small cells lung and pancreatic cancer.^12^, ^13^, ^14^, ^15^ However, the underlying mechanisms of WA’s effects on cancer are still unclear.

The endoplasmic reticulum (ER) stress response can be triggered by numerous conditions that cause imbalances in intracellular homeostasis, which in turn threaten proper cell functioning. In response, the ER stress response activates an adaptive effort to neutralize these threats for restoring homeostasis. When these countermeasures and severe imbalances persist, the ER stress response may initiate a pro‐apoptotic program to eliminate the faulty cells for the survival of the whole organism.^16^, ^17^ Otherwise, it is also reported that prolonged ER stress caused cell cycle arrest.^18^, ^19^ Choi et al demonstrated that WA induced CHOP‐mediated apoptosis.^20^ However, the relationship among ER stress, cell cycle arrest and apoptosis triggered by WA is still unclear.

In this study, we demonstrated that WA significantly inhibited the growth of U87 and U251 cells by inducing intrinsic apoptosis and arresting the cell cycle at the G2/M stage. WA also suppressed the growth of GBM cells in vivo. Furthermore, we have revealed a new mechanism for WA‐induced apoptosis and cell cycle arrest through the ATF4‐ATF3‐CHOP axis. These results suggested that WA inhibited the growth of GBM by a novel mechanism.

## 2 MATERIALS AND METHODS

### 2.1 Chemical agents and antibodies

Withaferin A (BC grade, >98%) was purchased from Sangon Biotech (Shanghai, China).3‐(4,5‐dimethyl‐2‐thiazolyl)‐2,5‐diphenyl‐2‐H‐tetrazolium bromide (MTT), PI dyes, RNase, Penicillin and streptomycin were obtained from Sigma‐Aldrich. Emricasan was purchased from Targetmol. Foetal bovine serum (FBS) and Dulbecco's Modified Eagle Medium (DMEM) were bought from Gibco. The goat‐anti‐rabbit secondary antibody, rabbit monoclonal Bad, GAPDH, phospho‐Ser (112) Bad, Bim, Bak, Bcl‐xL, caspase 3/7/9, cleaved caspase 3/7/8/9, cleaved PARP1, CDK1, phospho‐Thr (161) CDK1, p21, XBP1‐s and ATF3 were obtained from Cell Signaling Technology. Rabbit polyclonal Bax, Bcl‐2, cyclin A/B, p53, HMOX1, DNJB1, ATF6, ATF4, GRP78, CHOP and PPP1R15A were purchased from Proteintech. The details for ID code and dilution of antibodies are listed in the supplemental materials (Table S1).

### 2.2 Cell culture

The human glioblastoma cell lines, U251 and U87, were purchased from the Cancer Institute & Hospital, Chinese Academy of Medical Sciences. The human normal astrocyte cells HA1800 were bought from *SCIENCELL*. Cells were maintained with DMEM supplemented with 10% FBS, streptomycin (100 μg/mL) and penicillin (100 U/mL). The cells were cultured at 37°C in an incubator with a humidified atmosphere of 5% CO_2_.

### 2.3 MTT assay

Cells were seeded into 96‐well plates at 5 × 10^3^ per well, cultured for 24 hours and treated with WA for the indicated time. After washed with PBS, cells were incubated with serum‐free DMEM containing 0.5 mg/mL MTT for 3‐4 hours. The supernatants were carefully removed and discarded, and the formazan was dissolved in 100 μL dimethyl sulfoxide, followed by measurement with a SpectraMax M5 plate reader (Molecular Devices) at 570 nm.

### 2.4 Apoptosis assay

The apoptosis rate of cells was determined by using a Annexin V‐FITC kit from Beyotime Biotechnology. After treatment with WA for the indicated time, cells were harvested and washed with PBS. The cell pellets were resuspended in an annexin v‐FITC buffer and incubated on ice for 7 minutes. Then, PI was added into the mixture, and after 3 minutes, cell apoptosis was measured by flow cytometry (FACS Calibur, BD BioSciences). The results were analysed by FlowJo 7.6 software.

### 2.5 JC‐1 assay

The JC‐1 dye assay was conducted using JC‐1 kits purchased from Beyotime Biotechnology according to manufacturer's instructions. Briefly, after treated with WA for the indicated time, cells were harvested and washed three times with PBS. The cells were incubated in JC‐1 buffer for 20 minutes at 37 °C. After washing once with PBS, the cells were resuspended in PBS and measured by flow cytometry. Healthy mitochondria show red florescence, while damaged organelles fluoresce green.

### 2.6 Cell cycle assay

Cell cycle assays were performed by Flow cytometry. After treatment with WA for the indicated time, the cells were harvested and fixed with 75% ethanol at 4 °C for 24 hours, followed by staining in PI (200 μg/mL RNase, 50 μg/mL PI and 0.1% (v/v) Triton X‐100 in PBS) for 30 minutes. Cell cycle stage was determined by Flow cytometry.

### 2.7 RNA‐seq data analysis

Cells were treated with 3 μmol/L WA for 12 hours and harvested in TRizol for RNA isolation and sequencing. Initial isolates were checked for quality by FastQC software and filtered to remove low‐quality cells using default parameters and specifying a minimum length of 50. Processed reads were then aligned to the *Homo sapiens* genome assembly with Cuffmerge software. The levels of mRNA were evaluated by fragments per kilo bases per million fragments (FPKM) using Cuffquant and Cuffnorm software. The sample correlation analysis was performed using the Pearson coefficient. Cuffdiff software was used to analyse the differential expression, and the default screen standard for differential gene was |log_2_FC| ≥ 1 and *P* value ≤ .05. Herein, Log_2_FC means Log_2_(fold change). The two following comparisons were analysed: U87 treated with 3 μmol/L WA vs U87 without treatment and U251 treated with 3 μmol/L WA vs U251 without treatment. Moreover, log_2_FC ≥ 1 represents up‐regulated genes while log_2_FC ≤ −1 indicates down‐regulated genes. The gene ontology enrichment analysis was performed using DAVID Bioinformatics Resources 6.8 (https://david.ncifcrf.gov/).

### 2.8 RNA extraction, cDNA synthesis and qRT‐PCR

Cells were treated with 3 μmol/L WA for the indicated times and harvested in Trizol. After mixing with 1/5 volume of chloroform, the mixture was centrifuged at 13 201 *g* for 15 minutes and supernatants were transferred into new, clear centrifuge tubes. An equal volume of isopropanol was added to each supernatant and gently mixed. After incubation at room temperature for 30 minutes, the mixture was centrifuged at 13 201 *g* for 15 minutes. The pellets were washed once with 75% ethanol and dissolved in RNase‐free water at an appropriate volume. After RNA quantification, cDNA was synthesized using PrimeScript™ RT 1st Master Mix according to the manufacturer's instructions. Quantitative real‐time RT‐PCR (qRT‐PCR) was performed using TB Green® Premix Ex Taq^TM^ II (Tli RNaseH Plus). The primers used are listed in the supplemental materials section (Table S2). GAPDH served as internal control.

### 2.9 siRNA transfection

siRNA duplexes were obtained from Genepharm and used to transfect cells according to the recommended procedure.^21^ Briefly, U251 cells were seeded into 6‐well plates and cultured for 24 hours at 37 °C. Cells were transfected with 100 pmol of the indicated siRNA using Lipofectamine 2000 according to the manufacturer's directions. After 48 hours, the cells were incubated with 3 μmol/L WA for 24 hours. The sequences of siRNAs used in this study are listed in supplemental materials (Table S3).

### 2.10 Western blotting

After the indicated treatments, cells were harvested and resuspended in RIPA buffer for protein extraction. Protein concentration was determined by using a BCA assay kit from APPLYGEN. Aliquots of 80 to100 μg of protein were separated by 10% SDS‐PAGE and then transferred onto PVDF membranes (Merck Millipore Ltd). The membranes were blocked with TBST containing 5% non‐fat milk at room temperature for 1 hours and incubated with the indicated antibodies at 4 °C overnight. Subsequently, the membranes were washed three times with TBST and incubated with secondary antibody conjugated to horseradish peroxidase at room temperature for 1 hour. Finally, the membranes were washed three times with TBST and incubated with ECL reagents. The membranes were examined using a chemiluminescence photodocumentation system photographed and quantitated.

### 2.11 Immunofluroescence

Immunofluorescence was performed according to a recommended procedure.^22^ U251 cells were seeded into a 96‐well black plate with clear bottom and cultured for 24 hours. After incubation with 3 μmol/L WA for the indicated time, the cells were fixed with 4% paraformaldehyde for 15 minutes at room temperature, washed with PBS and blocked with PBS containing 1% BSA (w/v) and 0.3% Triton X‐100 (v/v) for 1 hour at room temperature. Cells were then incubated with the indicated primary antibody diluted with PBS containing 1% BSA (w/v) and 0.3% Triton X‐100 (v/v) overnight at 4 °C. Cells were washed three times with PBS and incubated with the corresponding fluorescent secondary antibody for 2 hour at room temperature. After three washes with PBS, cells were stained with 10 μg/mL Hoechst 33342 for 30 minutes, washed with PBS and imaged by fluorescence microscopy (Nikon Eclipse Ti‐U).

### 2.12 Glioblastoma xenograft assay in nude mice

Four‐ to five‐week‐old athymic nude mice (16‐18 g) were provided by the Animal House in the Department of Animal Care Center at Institute of Materia Medica, Chinese Academy of Medical Science & Peking Union Medical College. The animals were housed at 24 °C with ad libitum access to food and water. All experimental procedures were carried out in accordance with institutional guidelines for the care and use of laboratory animals at the Institute of Materia Medica, Chinese Academy of Medical Science & Peking Union Medical College and the National Institutes of Health Guide for Care and Use of Laboratory Animals (publication no. 85‐23, revised 1985). An aliquot of 5 × 10^6^ U87 cells was subcutaneously injected into the right flank of each mouse. After tumours reached a mean group size of 40 to 50 mm^3^, mice were randomly distributed, five per group, for treatment with vehicle or WA (5 mg/kg). Tumour volume (mm^3^) was measured with a vernier caliper and calculated using the formula, (LW^2^)/2, where L and W represent length and width of the tumour. Drugs were dissolved in saline containing 15% PEG400 and injected into the tail vain every day for 27 days. The tumour growth inhibition rate was calculated using the formula IR (%) = (1 − TWt/TWc) × 100, where TWt and TWc are severally the mean tumour weight of treated and control groups.

### 2.13 Statistical analysis

All experiments were repeated three times. The data were expressed as mean ± SD. Statistical analysis was carried out using Origin 9.0 software, and comparisons of each group were made by one‐way analysis of variance (ANOVA) using SPSS 19 software. Results were considered statistically significant at *P* < .05.

## 3 RESULTS

### 3.1 WA‐induced apoptosis of U87 and U251 cells via the intrinsic pathway

**Figure 1:**
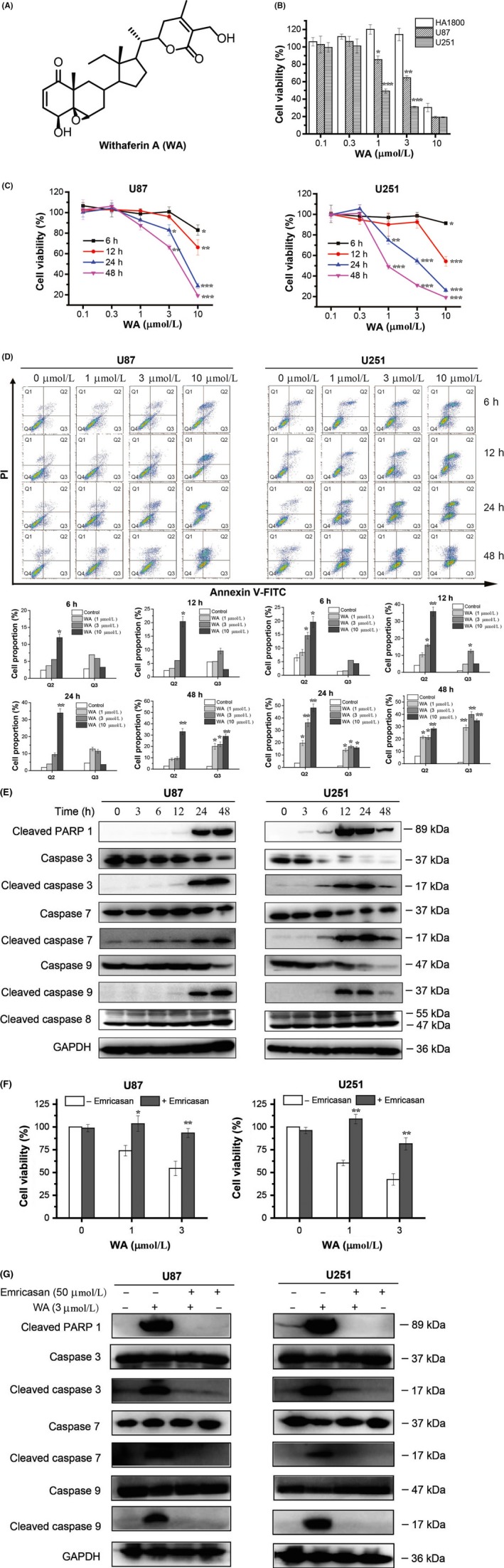
WA‐induced apoptosis of U87 and U251 cells via an intrinsic pathway. Cells were treated with WA at various concentrations for the indicated time, and harvested for MTT, Flow cytometry and Western blotting. Cells were pre‐treated with Emricasan (50 μmol/L) for 24 h and then treated with WA (1 or 3 μmol/L) for 48 h. A, The structure of Withaferin A (WA). B, The effects of WA on U87, U251 and HA1800 cells. *P < .05, **P < .01 and ***P < .01 represented significant differences between U87 and U251 cells vs HA1800 cells. C. Cell viability was measured by MTT assay. *P < .05, **P < .01 and ***P < .001 represented significant differences between the WA‐treated group and control group. D, The apoptotic rates were determined by Flow cytometry after staining for Annexin V conjugated with FITC and PI dyes. The Q1, Q2, Q3 and Q4 quadrants represented dead, late‐apoptotic, early‐apoptotic and normal cells, respectively. *P < .05 and **P < .01 indicated significant differences between the WA‐treated group and control group. E, Apoptotic proteins were identified by Western blotting. F, Cell viability treated with 3 μmol/L WA was measured by MTT assay after pre‐treated with Emiricasan (50 μmol/L) for 24 h. *P < .05 and **P < .01 represented the significant differences between the Emiricasan‐treated group and corresponding non‐Emiricasan‐treated group. G. Western blot was used to check the alteration of apoptotic proteins after pre‐treatment with Emricasan. WA, Withaferin A

To confirm the effects of WA (Figure 1A) on viability of GBM cells and normal astrocytes, an MTT assay was carried out on U87, U251 and HA1800 cells. As shown in Figure 1B, WA inhibited the growth of U87 and U251 more than normal astrocyte cells, HA1800, at the same concentration (1 or 3 μmol/L). The half‐maximal inhibitory concentration (IC_50_) in U87, U251 and HA1800 cells treated with WA for 48 hours was 4.61, 1.37 and 9.13 μmol/L, respectively. Moreover, WA inhibited the growth of U87 and U251 cells in a concentration‐ and time‐dependent manner (Figure 1C). To further investigate the effects of WA on the survival of U87 and U251 cells, the apoptosis rate was measured by Flow cytometry. The number of U87 or U251 cells in the Q2 and Q3 quadrants was augmented with increasing WA concentration and treatment time, indicating that WA‐induced apoptosis in a concentration‐ and time‐dependent manner (Figure 1D).

Once the apoptotic process is initiated, pro‐caspases will be cleaved to active forms and the commands of apoptosis, such as cleaving PARP to inhibit its activity to facilitate DNA damage will be executed.^23^, ^24^ As shown, cleaved PARP1 and caspase 3/7/9 were remarkably elevated in U87 and U251 cells after 24 hour treatment with 3 μmol/L WA (Figure 1E and Figure S1A). However, there was no change in cleaved caspase 8, which represents the extrinsic apoptotic pathway. These results suggested that WA induced apoptosis of U87 and U251 cells via the intrinsic pathway. After pre‐treatment with 50 μmol/L Emricasan (a pan‐caspase inhibitor) for 24 hours, cells were incubated with 1 or 3 μmol/L WA for 48 hours. Results showed that the effects of WA on cell viability and apoptosis were almost completely abrogated by Emricasan (Figure 1F and 1G, Figure S1B). Taken together, these results suggested that WA induced apoptosis of U87 and U251 cells via the intrinsic pathway.

### 3.2 WA‐induced apoptosis of U87 and U251 cells partly by up‐regulating expression of Bim and Bad

**Figure 2:**
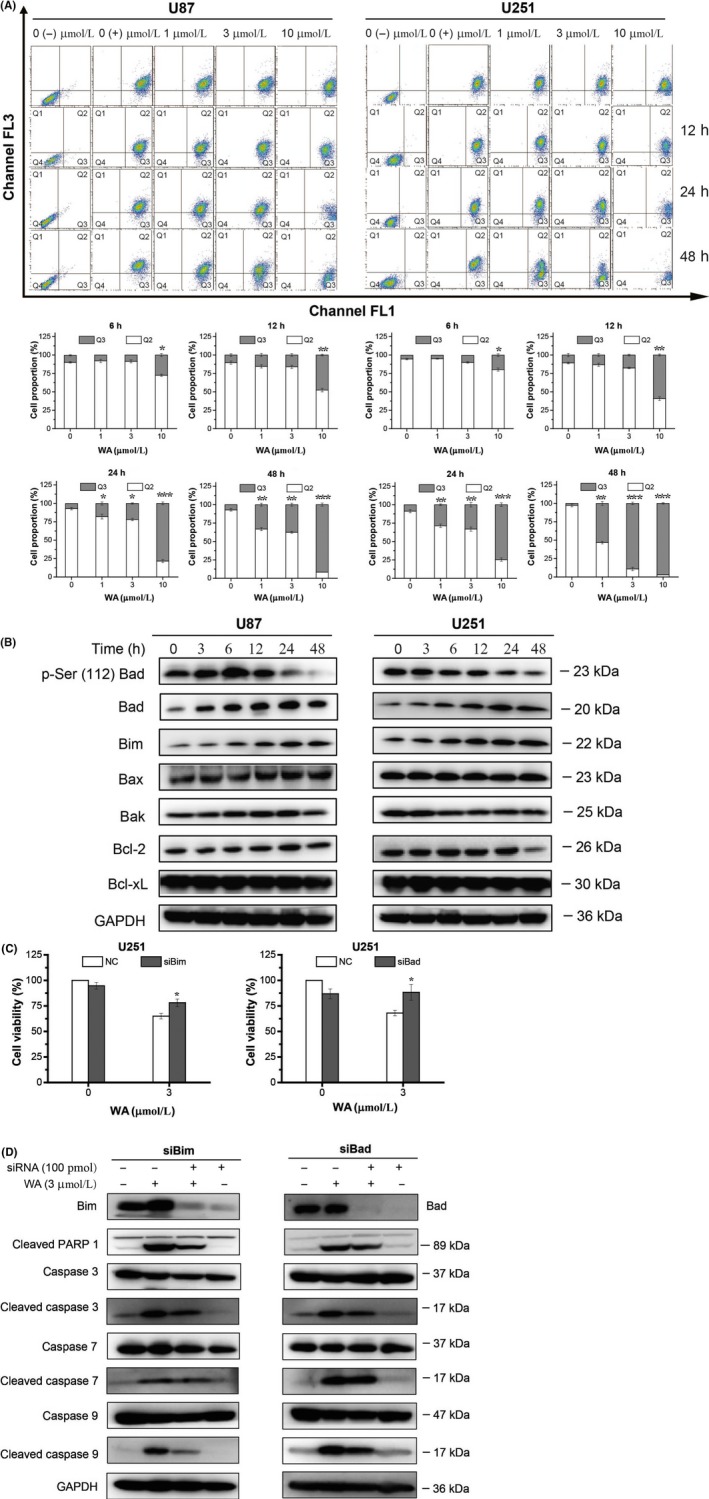
WA‐induced apoptosis of U87 and U251 cells partly by up‐regulating expression of Bim and Bad. After treatment with WA at the indicated concentrations for the indicated times, cells were separately harvested for Flow cytometry, Western blotting and MTT assay. For siRNA silencing, U251 cells were transfected with siRNA of Bim or Bad for 48 h and treated with 3 μmol/L WA for an additional 24 h. A, Mitochondrial membrane potential was determined by Flow cytometry after staining with JC‐1 dyes. The Q2 and Q3 quadrants represented normal cells and cells of decreased mitochondrial membrane potential, respectively. *P < .05, **P < .01 and ***P < .001 represented significant differences between the WA‐treated group and control group. B, Proteins regulating the intrinsic apoptotic pathway were detected by Western blotting. C, Cell viability of U251 was determined by MTT assay after transfection with siRNA of Bim or Bad. *P < .05 and **P < .01 represented significant differences between the siRNA‐treated group and corresponding non‐siRNA‐treated group. D, The changes of regulated proteins in intrinsic apoptotic pathway in U251 cells were determined by Western blotting after transfection with siRNA of Bim or Bad. WA, Withaferin A

To explore how WA activated the intrinsic apoptotic pathway, JC‐1 staining was carried out. As shown in Figure 2A, WA reduced mitochondria membrane potential in a concentration‐ and time‐dependent manner. WA treatment slightly changed the expression of the pro‐apoptotic proteins, Bax and Bak, as well as anti‐apoptotic proteins, Bcl‐2 and Bcl‐xL, while expression of the pro‐apoptotic proteins, Bim and Bad, was significantly increased after 6 hours (Figure 2B and Figure S2A). The phospho‐Ser^112^ level of Bad was decreased after treatment with WA for 24 hours (Figure 2B and Figure S2A). Thus, Bim and Bad may be the key regulators that initiated the intrinsic apoptotic pathway. To confirm this hypothesis, the expression of Bad and Bim was knocked down by corresponding siRNA in U251 cells. As expected, the viability of WA‐treated U251 was increased by siRNAs of Bad or Bim (Figure 2C). Similarly, the alterations of cleaved caspase 3 and 9 caused by WA in U251 cells were also partly reversed by siRNAs of Bad or Bim (Figure 2D and Figure S2B). Together, these results suggested that the apoptosis induced by WA was partly mediated by up‐regulating the expression of Bad and Bim.

### 3.3 Cell cycle was arrested at the G2/M phase by WA through p53‐independent p21 up‐regulation

**Figure 3:**
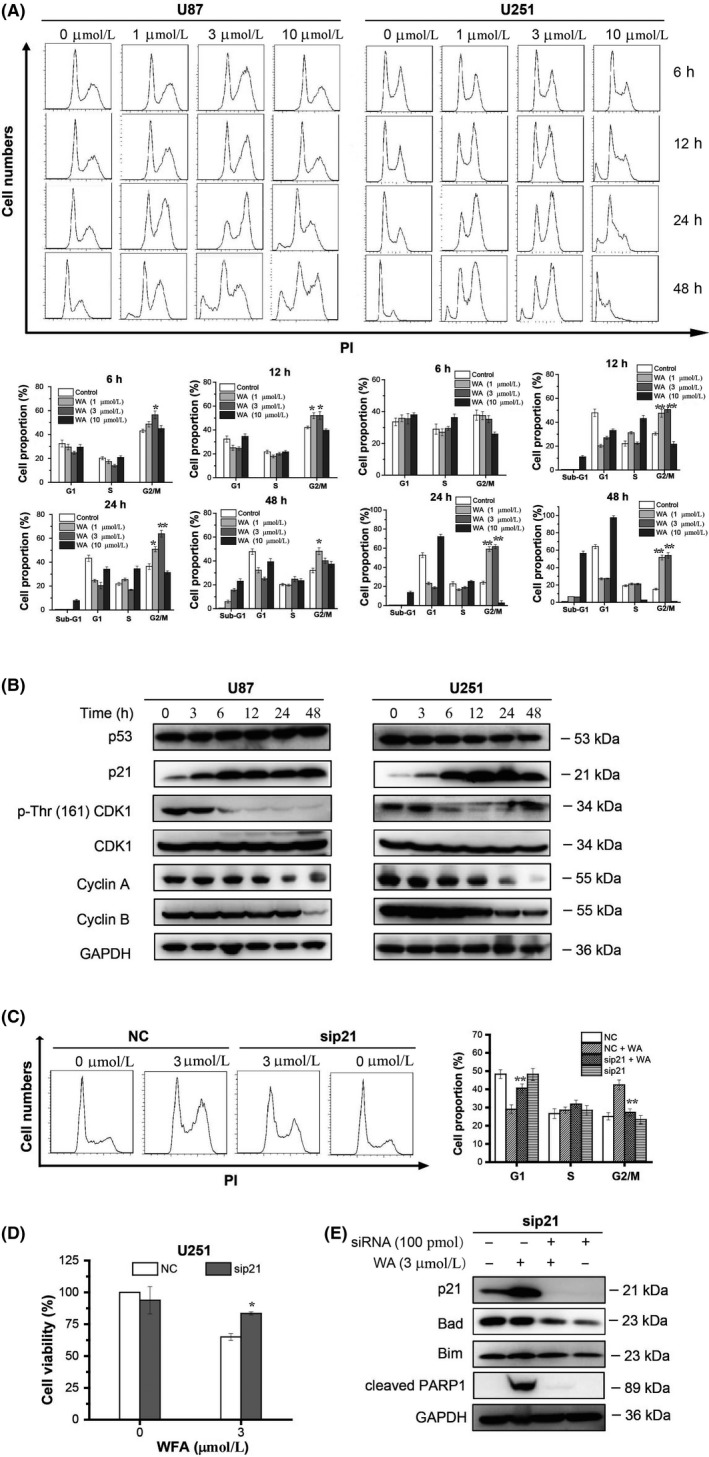
The cell cycle was arrested at the G2/M phase by WA through p53‐independent p21 up‐regulation. Cells were treated with WA at the indicated concentration for the indicated time and then assayed by flow cytometry and Western immunoassay. For siRNA silencing, U251 cells were transfected with siRNA of p21 for 48 h then treated with 3 μmol/L WA for 24 h longer, and finally analysed by MTT, Flow cytometry and Western blotting. A, Cell cycle was determined by Flow cytometry after staining with PI. *P < .05, and **P < .01 represented significant differences of cells at G2/M phase vs control group. B, Proteins regulating the G2/M phase were detected by Western blotting. C, Cell cycle analysis after p21 knockout was performed by Flow cytometry. **P < .01 represented significant differences between the sip21‐treated group and NC‐treated group. D, Cell viability of U251 was measured by MTT assay after transfection with siRNA of p21. *P < .05 represented significant differences between the siRNA‐treated group and the corresponding non‐siRNA‐treated group. E, The changes of Bim, Bad and cleaved PARP1 proteins in U251 cells were checked by Western blotting after transfection with siRNA of p21. WA, Withaferin A

To determine the effects of WA on cell cycle in U87 and U251 cells, PI was used to stain the cells after WA treatment for 6, 12, 24 and 48 hours. As shown in Figure 3A, the number of cells at G2/M phase was significantly increased after 12 hours incubation with 1 or 3 μmol/L WA. WA at a concentration of 10 μmol/L had almost no effect on cell cycle, but caused an increase of cells at sub‐G1, which represented apoptotic cells. To determine whether expression of CDK1, cyclin A and cyclin B, which are responsible for G2/M regulation, was affected by WA incubation, we measured protein levels by Western blotting. The expression of cyclin B was reduced beginning at 48 hours in U87 or at 12 hours in U251 cells, while cyclin A was reduced from 24 hours in both cell types (Figure 3B and Figure S3A). The total CDK1 expression was not affected by WA treatment, but the levels of phospho‐Thr^161^ CDK1 were significantly decreased from 6 hours in both cell types, indicating that the active site of CDK1 was inhibited (Figure 3B and Figure S3A). The cyclin‐dependent kinase inhibitor, p21, suppresses the phosphorylation of CDK1 at Thr^161^, and we thus measured its expression by Western blotting. We found that p21 was up‐regulated in both cell types after treatment with 3 μmol/L WA for 6 hours (Figure 3B and Figure S3A), implying that p21 was involved in the G2/M arrest induced by WA. Intriguingly, the upstream regulator of p21, p53, remained unchanged after 3 μmol/L WA treatment, which indicated the existence of a p53‐independent pathway for regulation of p21 expression. When expression of p21 in U251 cells was knocked down by p21 siRNA, the G2/M phase arrest caused by WA was partly reversed, further confirming that p21 played an important role in WA‐induced arrest of cell cycle (Figure 3C). Interestingly, the loss of cell viability caused by WA was significantly restored by p21 siRNA (Figure 3D). Thus, we further investigated the relationship between p21 and Bad or Bim. The expression of Bad rather than Bim was remarkably reduced by p21 siRNA in U251 cells (Figure 3E and Figure S3B). Furthermore, p21 knockdown reduced the levels of cleaved PARP1, an apoptotic marker (Figure 3E and Figure S3B). Taken together, all these results suggested that WA induced apoptosis by regulating the p21‐Bad pathway.

### 3.4 Expression of HMOX1, DNJB1 and ATF3 was induced by WA at an early stage of treatment

**Figure 4:**
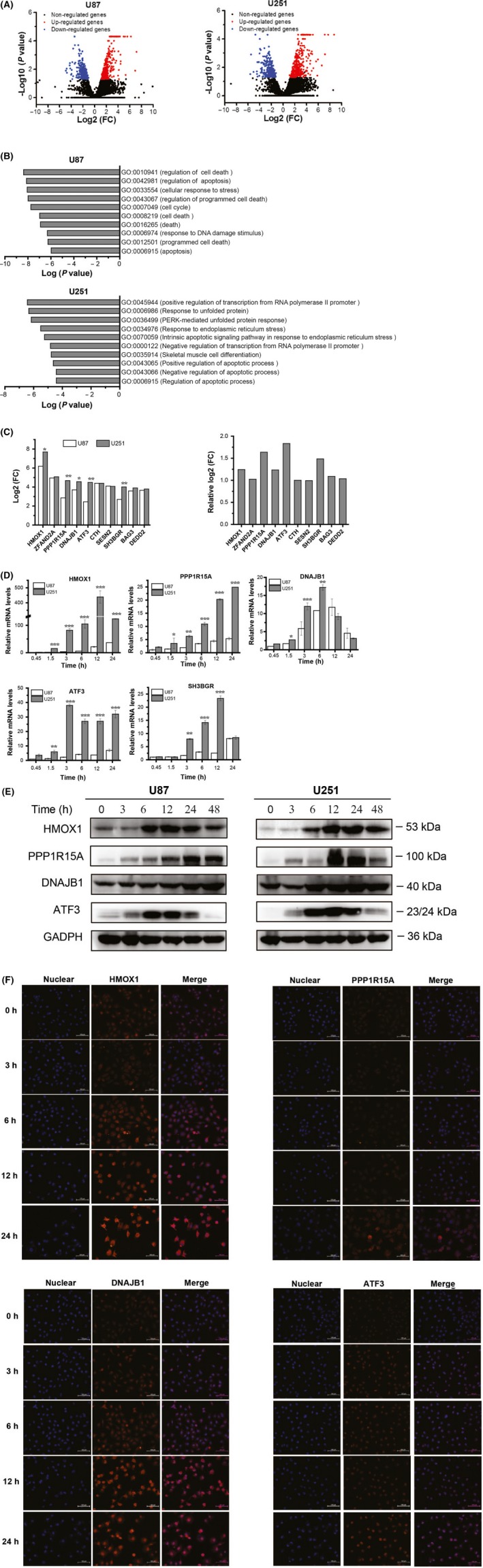
Expression of HMOX1, DNJB1 and ATF3 was induced by WA at an early stage of treatment. After treatment with 3 μmol/L WA for 12 h, cells were harvested and analysed by RNA‐seq. A, The differential gene expression caused by WA in U251 and U87 cells was determined by RNA‐seq. B, The cellular signalling pathways triggered by WA in U251 and U87 cells were obtained by GO enrichment at DAVID website. C, The top ten differentially expressed genes in both U251 and U87 cells are listed. *P < .05 and **P < .01 represented significant differences between U251 cells and U87 cells at the same gene. D, The changes of HMOX1, PPP1R15A, DNJB1, ATF3 and SH3BGR in U251 and U87 cells were detected at the mRNA level after WA treatment for 0.45, 1.5, 3, 6, 12 and 24 h. * *P* < .05, **P < .01 and ***P < .001 represented significant differences between U251 cells and U87 cells. E, The changes in HMOX1, PPP1R15A, DNJB1 and ATF3 in U251 and U87 cells were detected from expression levels after WA treatment for 3, 6, 12, 24 and 48 h. F, Immunofluorescence was used to observe the nuclear translocation of HMOX1, PPP1R15A, DNJB1 and ATF3 in U251 and U87 cells caused by WA. WA, Withaferin A

To explore the early responses to WA, the cells were treated with 3 μmol/L WA for 12 hours and used to perform RNA sequence analysis. After analysing the data from RNA‐seq, we found 276 up‐regulated genes and 264 down‐regulated genes in U87 cells, as well as 374 up‐regulated genes and 264 down‐regulated genes in U251 cells (Figure 4A). All the differentially expressed genes were uploaded on the DEVID website for GO enrichment to search for involvement of potential cell signalling pathways. The top ten GO enrichments are listed in Figure 4B. These GO enrichments in U87 cells mainly focused on cell death, apoptosis, cell response to stress, programmed cell death and cell cycle. However, the top five signalling pathways in the U251 cells were concentrated in (a) positive regulation of transcription from the RNA polymerase II promoter, (b) response to unfolded proteins, (c) PERK‐mediated unfolded protein response, (d) response to endoplasmic reticulum stress and (e) intrinsic apoptotic signalling pathway in response to ER stress. The top ten genes in both U87 and U251 cells with the greatest significant differences are listed at Figure 4C. Because U251 cells were more sensitive to WA than U87 cells, the genes with higher changes in U251 than U87 were much more likely to be targets for WA. Therefore, HMOX1, PP1R15A, DNAJB1, ATF3 and SH3BGR were tested by performing qRT‐PCR. The results showed that mRNA levels of HMOX1, PP1R15A, DNAJB1 and ATF3 were remarkably increased beginning as early as 1.5 hours treatment with WA and were augmented in a time‐dependent manner (Figure 4D). Next, we analysed the expression levels of HMOX1, PP1R15A, DNAJB1 and ATF3 by Western immunoblotting and their intracellular nuclear translocation in U251 cells by immunofluorescence. The results indicated that HMOX1and ATF3 were significantly elevated after 6 hours treatment with WA in both U87 and U251 cells whereas the expression of DNAJB1 was only increased in U251 cells at an early stage (Figure 4E and Figure S4). In addition, PP1R15A was also increased by WA after 12 hours treatment. The results of immunofluorescence confirmed the above results and showed that there was no nuclear translocation of HMOX1, PP1R15A, DNAJB1 and ATF3 occurring in U251 cells (Figure 4F).

### 3.5 WA‐induced apoptosis and G2/M arrest of GBM cells by ATF4‐ATF3‐CHOP axis

**Figure 5:**
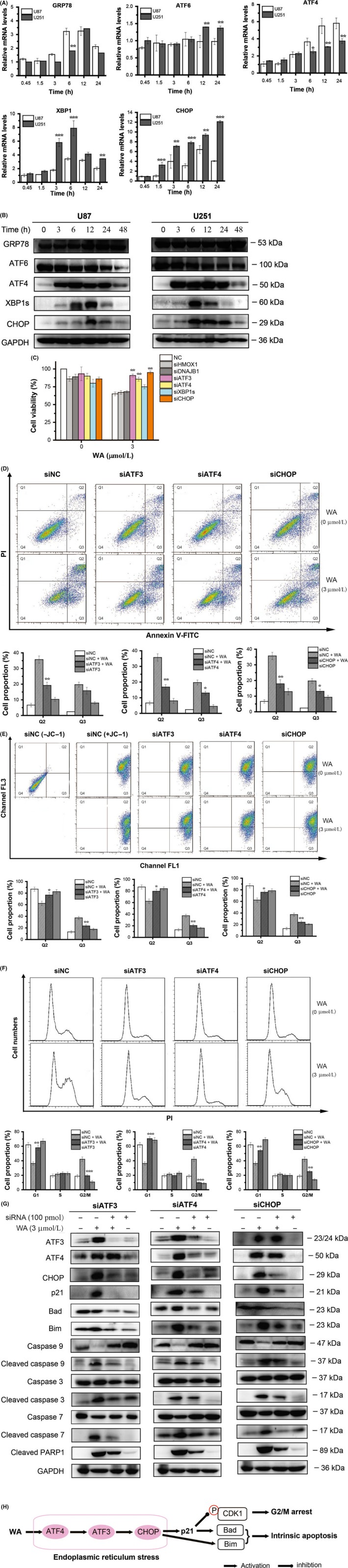
WA‐induced apoptosis and G2/M arrest of GBM cells by ATF4‐ATF3‐CHOP axis. Cells were treated with 3 μmol/L WA for the indicated time and harvested for qRT‐PCR and Western blotting. For siRNA silencing, U251 cells were separately transfected with siRNAs of HMOX1, DNAJB1, ATF3, ATF4, XBP1 and CHOP for 48 h, then treated with 3 μmol/L WA for an additional 24 h, and finally analysed by MTT, Western blotting and Flow cytometry. A, Genes associated with ER stress were determined by qRT‐PCR. *P < .05, **P < .01 and ***P < .001 represented significant differences between U251 cells and U87 cells. B, Proteins associated with ER stress were identified by Western blotting. C, Cell viability of U251 cells treated by 3 μmol/L WA was measured by MTT assay after transfection with siRNAs of HMOX1, DNAJB1, ATF3, ATF4, XBP1 and CHOP, respectively. **P < .01 represented significant differences between the siRNA‐treated group and the corresponding negative control group. D, Apoptosis of U251 treated with 3 μmol/L WA was measured by Fow cytometry after transfection with siRNAs of ATF3, ATF4 and CHOP, respectively. *P < .05 and **P < .01 represented significant differences between the siRNA‐treated group and the corresponding negative control group. E, Mitochondrial membrane potentials of U251 treated with 3 μmol/L WA were measured by Flow cytometry after transfection with siRNAs of ATF3, ATF4 and CHOP, respectively. *P < .05 and **P < .01 represented significant differences between the siRNA‐treated group and the corresponding negative control group. F, The cell cycle stage of U251 treated with 3 μmol/L WA was determined by Flow cytometry after transfection with siRNAs of ATF3, ATF4 and CHOP, respectively. **P < .01 and ***P < .01 represented significant differences between the siRNA‐treated group and the corresponding negative control group. G, The changes of p21, Bim, Bad and cleaved‐PARP1, caspase 3/7/9 and cleaved caspase 3/7/9 proteins in U251 cells treated with 3 μmol/L WA were determined by Western blotting after transfection with siRNAs of ATF3, ATF4 and CHOP, respectively. H, The overall mechanism of WA activity in GBM cells. WA, Withaferin A

To investigate the mechanism underlying the effects of WA on GBM cells, RNA‐seq was carried out to determine the transcriptional changes in cells caused by WA. The results from RNA‐seq suggested that the gene expression response to ER stress might play an important role in the pharmacological effects of WA. We validated the changes of target proteins including GRP78, ATF6, ATF4, XBP1‐s (the active form of XBP1) and CHOP at the mRNA and protein levels. Our results demonstrated that WA triggered up‐regulation of ATF4, XBP1 and CHOP, but had little effect on ATF6 and GRP78 at the levels of both mRNA and protein (Figure 5A and 5B, Figure S5A). WA increased the expression of ATF4, followed by XBP1‐s and CHOP (Figure 5B and Figure S5A). When expressions of HMOX1, DNAJB1, ATF3, ATF4, XBP1 and CHOP were knocked down by corresponding siRNAs, the viability of U251 cells inhibited by 3 μmol/L WA was significantly (*P* < .01) recovered by ATF3, ATF4 and CHOP siRNAs (Figure 5C). The induction of apoptosis by WA and its (Figure 5D) effect on mitochondrial membrane potential (Figure 5E) and cell cycle (Figure 5F) in U251 cells were counteracted by separately knocking down ATF3, ATF4 or CHOP. The positive effects of WA in p21, Bad, Bim, cleaved caspase 3/7/9 and cleaved PARP1 were also diminished by all three siRNA duplexes (Figure 5G and Figure S5B). Those results suggested that ATF3, ATF4 and CHOP may be the pivotal regulators for WA‐induced apoptosis and G2/M arrest. The results shown in Figure 5E demonstrated a logical relationship among ATF3, ATF4 and CHOP—ATF4 positively regulated ATF3, subsequently modulating the expression of CHOP.

### 3.6 WA inhibited the growth of U87 xenograft in nude mice

**Figure 6:**
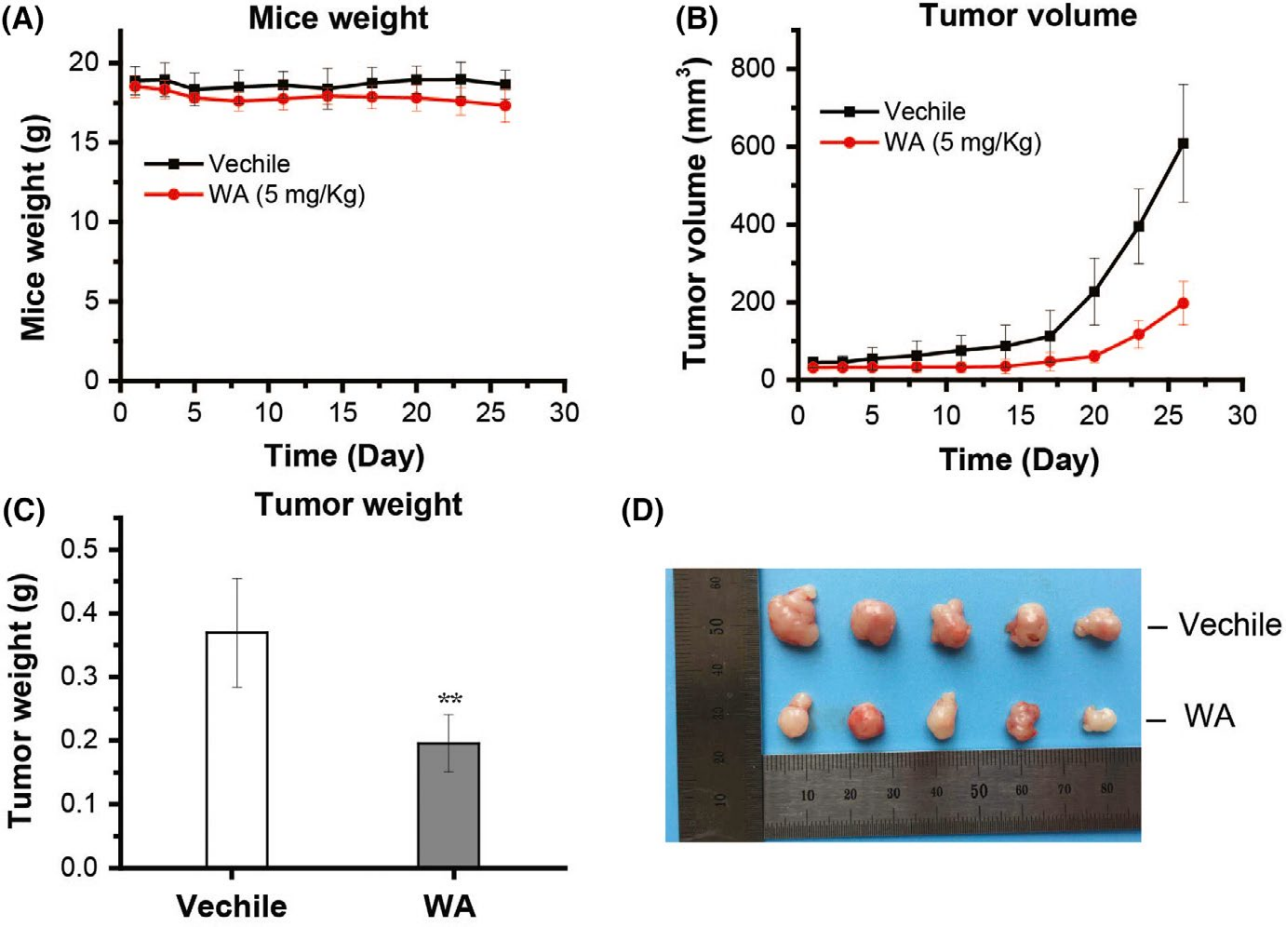
WA inhibited the growth of subcutaneous U87 xenografts. U87 cells (5 × 10^6^) were subcutaneously injected into the right flank of nude mice. After the tumour reached a volume of 40‐50 mm^3^, the mice were injected with vehicle or 5 mg/kg WA in the tail vein every day for 1 month. A, WA had no effect on the weight of the mice. B, WA reduced the tumour volume. C, WA decreased the tumour weight. **P < .01 represented significant difference between the WA group and the vehicle group. D, The photographs of tumours were collected from different groups of mice at the end of treatment (day 27). WA, Withaferin A

To check whether WA had anti‐tumour effects in vivo, U87 xenografts in nude mice were used. In this study, U87 cells (5 × 10^6^) in PBS were injected subcutaneously into the right flank. As shown in Figure 6, the weight of the mice was slightly decreased in both experimental and control groups while the volume and weight of tumours in the group injected with 5 mg/Kg WA were significantly smaller than in the control group injected with only saline. These results demonstrated that WA treatment could inhibit the growth of U87 xenografts in mice.

## 4 DISCUSSION

Elucidation of the mechanism underlying the effects of WA on tumour cells is important for optimization of WA‐based regimens for prevention and/or treatment of cancer, and discovering new targets for anti‐cancer drug development. It is known that the same drug may trigger different signalling pathway in different kinds of cells and thereby exert different pharmacological effects. Herein, we evaluated the efficacy of WA against GBM, which is one of the most lethal malignant tumours originating in the central nervous system, in vivo and in vitro. The results showed that WA exerted more inhibition on U251 and U87 cells than on the normal astrocyte line, HA1800 (Figure 1B), as well as on a U87 xenograft implanted subcutaneously in nude mice (Figure 6), suggesting that WA may be an effective drug for GBM therapy.

Overwhelming evidence indicated that WA induced apoptosis of cancer cells. Lee et al showed that WA promoted TRAIL‐induced apoptosis of the human renal cancer cells (Caki) through up‐regulation of death receptor 5 and down‐regulation of c‐FLIP.^25^ Hahm et al indicated that WA activated Bax and Bak to induce the apoptosis of MDA‐MB‐231 and MCF‐7 cells.^14^ Stan et al addressed the mechanism by which WA caused Bim‐dependent apoptosis.^26^ Our results showed that WA induced apoptosis of GBM cells by the intrinsic rather than the extrinsic pathway (Figure 1). Furthermore, our data showed that expression of Bax, Bak, Bcl‐2 and Bcl‐xL was not affected by WA in GBM cells (Figure 2B and Figure S2A). Besides Bim which played an important role in intrinsic apoptotic pathway induced by WA, the expressions of Bad and dephosphorylated Ser^112^ of Bad were induced to promote apoptosis of GBM cells (Figure 2C and 2D, Figure S2B), which was first reported by our group.

It is well known that WA can cause cell cycle arrest at the G2/M phase in cancer cells.^10^, ^11^, ^27^ Our data confirmed that WA was able to arrest the cell cycle of U87 and U251 cells at the G2/M phase (Figure 3A). Previous studies reported that the mechanisms involved in effects of WA on cell cycle were accumulation of Tyr^15^ phosphorylated (inactive) CDK1 via a decrease in levels of cell division cycle 24B (Cdc24B) and/or Cdc24C proteins,^27^ p51‐dependent p21 up‐regulation 28 and down‐regulation of cyclins (E2, A and B1).^29^ In our study, the results showed that cyclin A and B1 were down‐regulated in the later stages of WA treatment (24 and 48 hours). However, significant up‐regulation of p21 appeared after 6 hours of WA treatment. Moreover, silencing of p21 with siRNA partly reversed the G2/M phase arrest induced by WA (Figure 3C). In addition, WA did not change the level of p53 but dephosphorylated CDK1 at Thr^161^ (Figure 3B and Figure S3A). These results suggested that WA induced arrest of G2/M phase by dephosphorylating CDK1 at Thr^161^ via a p53‐independent p21 up‐regulation. More interestingly, p21 knockdown contributed to a decrease in the levels of cleaved PARP1 and Bad rather than Bim, suggesting that WA may induce GBM apoptosis via the p21‐Bad axis (Figure 3D and 3E, Figure S2B).

One important aim of our study was to explore the early protein response to WA. Thus, RNA‐seq analysis was carried out and the results implied that ER stress may be a breakthrough point. The ER is an organelle that is responsible for protein folding and assembly, and exquisitely sensitive to alterations in homeostasis.^30^ Under an unfolded protein response (UPR), three primary ER‐localized protein stress sensors (IRE1α, PERK and ATF6) were activated.^31^, ^32^, ^33^ The activated IRE1α can function as an endoribonuclease to initiate removal of a 26 nucleotide intron from the mRNA encoding XBP1 to form XBP1‐s,^34^, ^35^, ^36^ while the PERK‐mediated pathway involved up‐regulation of ATF4 and CHOP.^32^, ^37^, ^38^ ATF3 belongs to the ATF/cAMP‐response element‐binding protein family of transcription factors and plays an important role in ER stress.^39^, ^40^ It was reported that ATF3 and ATF4 form the complex to improve the transcription of CHOP for induction of apoptosis.^41^, ^42^ Our work showed that treatment with WA up‐regulated expression of ATF4, and then ATF3, CHOP as well as XBP1‐s (Figure 4 and Figure 5). Knockdown of only one of the associated proteins, ATF4, ATF3 and CHOP, could significantly restore the inhibition of U251 cells caused by WA (Figure 5C). Furthermore, blocking their expression significantly reduced the inhibition of WA in apoptosis (Figure 5D), mitochondrial membrane potential (Figure 5E) and cell cycles (Figure 5F), as well as expression levels of p21, Bad, Bim, cleaved PARP1 and caspase 3/7/9 (Figure 5G and Figure S5B). Our work also revealed that treatment with WA triggered the up‐regulation of ATF4, which induced expression of ATF3, and then positively modulated CHOP (Figure 5G and Figure S5B).

In summary, we demonstrated that WA exhibited significant growth inhibitory effect against GBM cells in vitro and in vivo, which is associated with a Bim‐ and Bad‐dependent intrinsic apoptotic pathway and arrest of G2/M phase induced by p21 up‐regulation. The novel upstream regulator of Bim, Bad and p21 discovered in our study is the ATF4‐ATF3‐CHOP axis. The overall mechanism is illustrated in Figure 5H. Our study provides important mechanistic insights into using WA in future clinical scenarios where it may synergize with current therapeutic strategies.

## CONFLICT OF INTEREST

The authors declare no conflicts of interest that pertain to this work.

## AUTHOR CONTRIBUTIONS

Qin Tang designed the research methods, performed the experiments, analysed the data and drafted the manuscript. Liwen Ren and Jinyi Liu participated in flow cytometry experiments and data collection. Xiangjin Zheng and Wan Li collected tumour tissues. Jinhua Wang and Guanhua Du designed the research and revised the manuscript. All authors have read and approved the final manuscript.

## Data Availability

The data that support the findings of this study are available from the corresponding author upon reasonable request.
