# Correction to “Withaferin A triggers G2/M arrest and intrinsic apoptosis in glioblastoma cells via ATF4‐ATF3‐CHOP axis”

**DOI:** 10.1111/cpr.13706

**Published:** 2024-06-26

Tang Q , Ren L , Liu J , et al. Withaferin A triggers G2/M arrest and intrinsic apoptosis in glioblastoma cells via ATF4‐ATF3‐CHOP axis. Cell Prolif. 2020;53:e12706.31642559 10.1111/cpr.12706PMC6985693

In Figure 4F, the 3 h and 24 h figures of Nuclear of DNAJB1 were not matched with the corresponding Merge figures. The corrected figure is below:

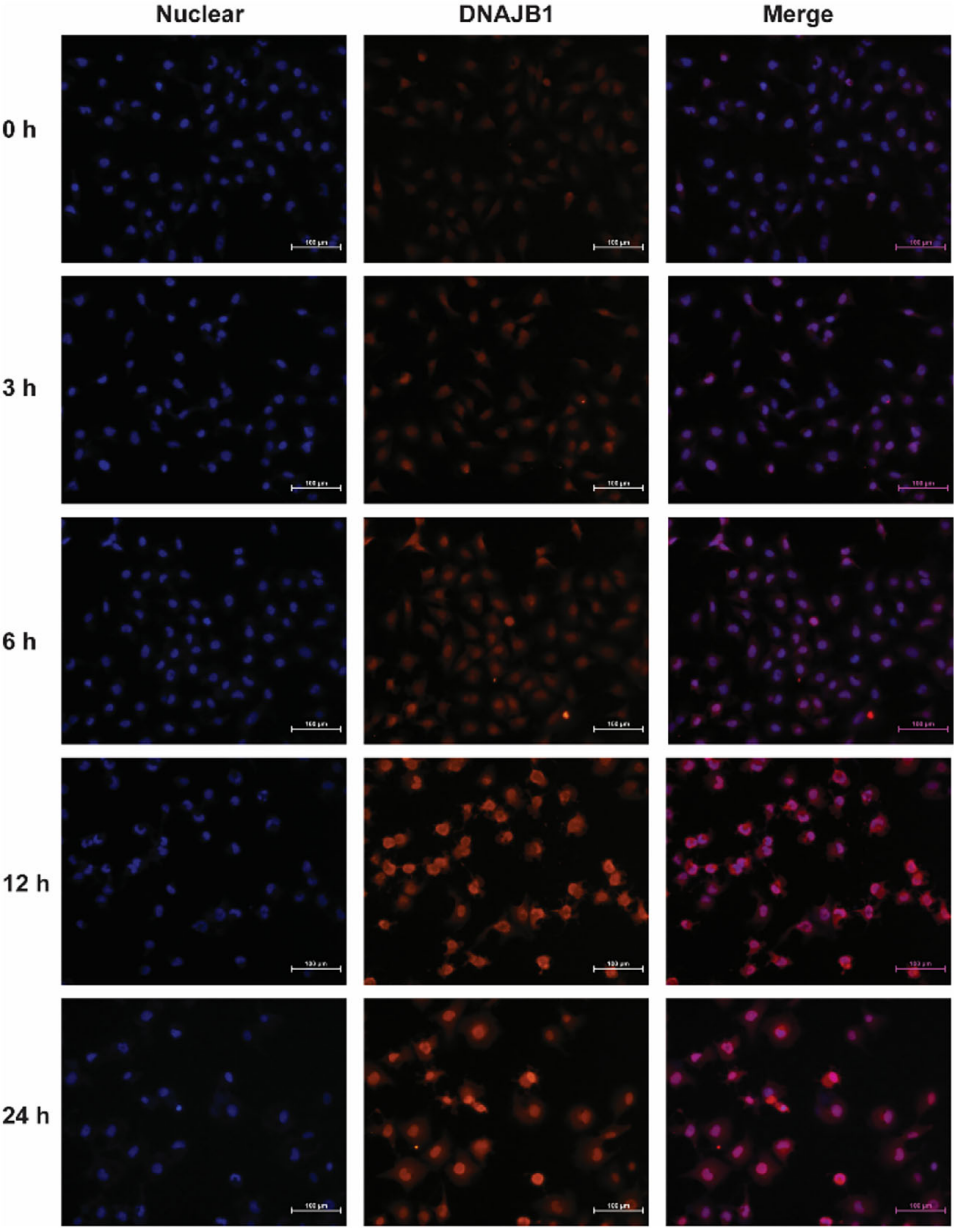

We apologize for this error.

