# Supplementary figures and images for: Withaferin A triggers G2/M arrest and intrinsic apoptosis in glioblastoma cells via ATF4‐ATF3‐CHOP axis
Source: Cell Prolif. 2019 Oct 23;53(1):e12706. doi: 10.1111/cpr.12706 (PMC6985693; doi:10.1111/cpr.12706)

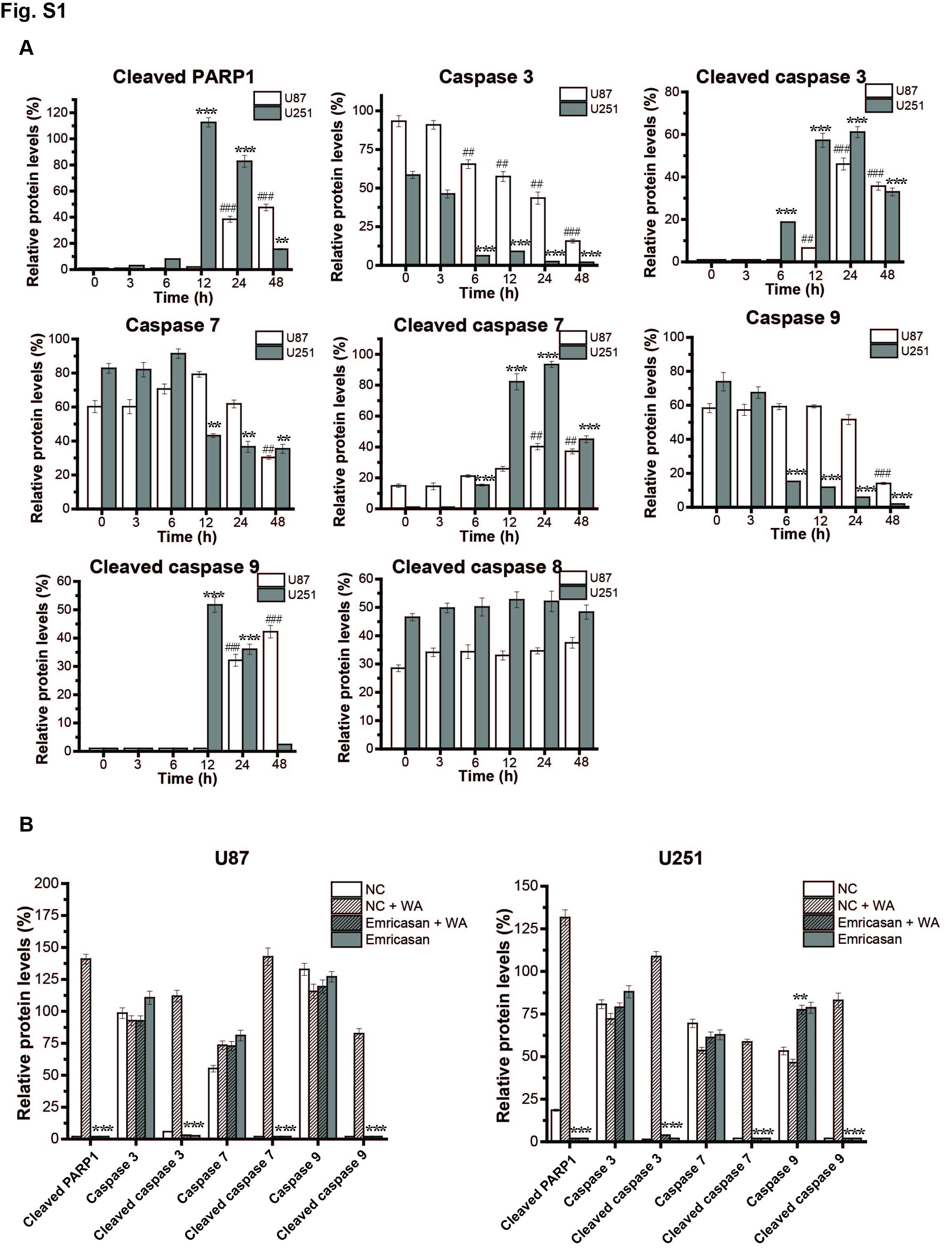

Supplement: Supplementary file 1 [file CPR-53-e12706-s001.jpg]

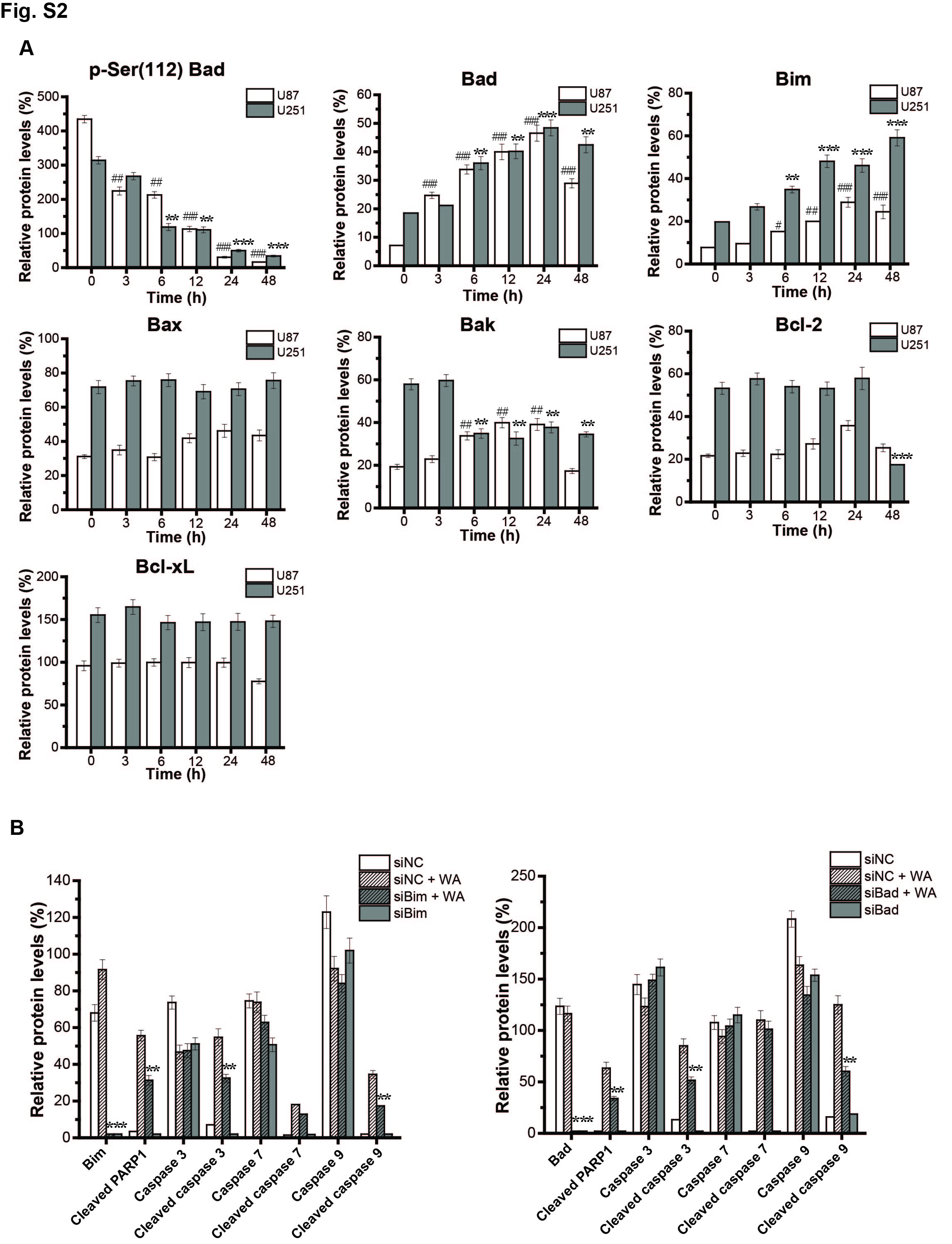

Supplement: Supplementary file 2 [file CPR-53-e12706-s002.jpg]

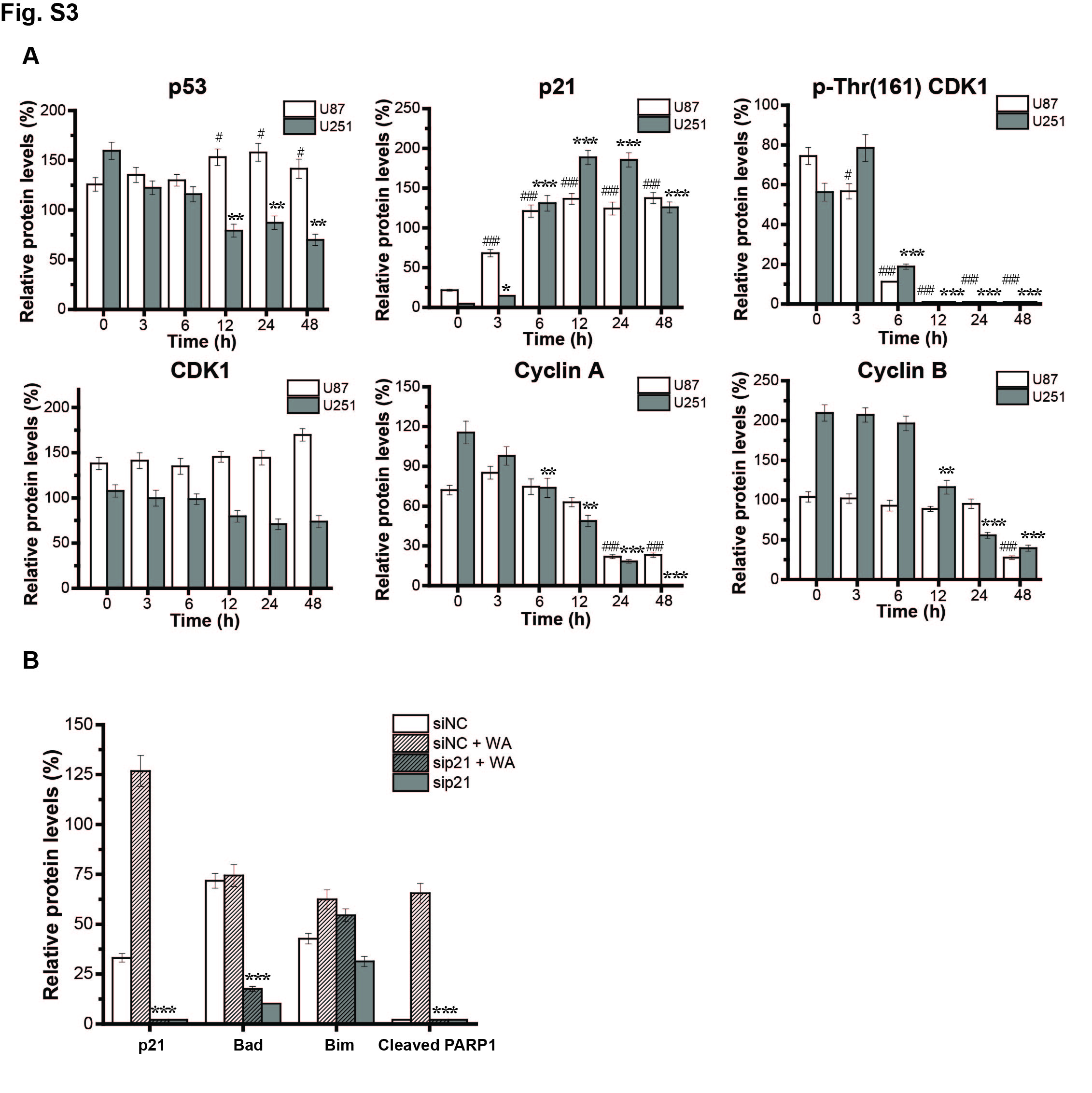

Supplement: Supplementary file 3 [file CPR-53-e12706-s003.jpg]

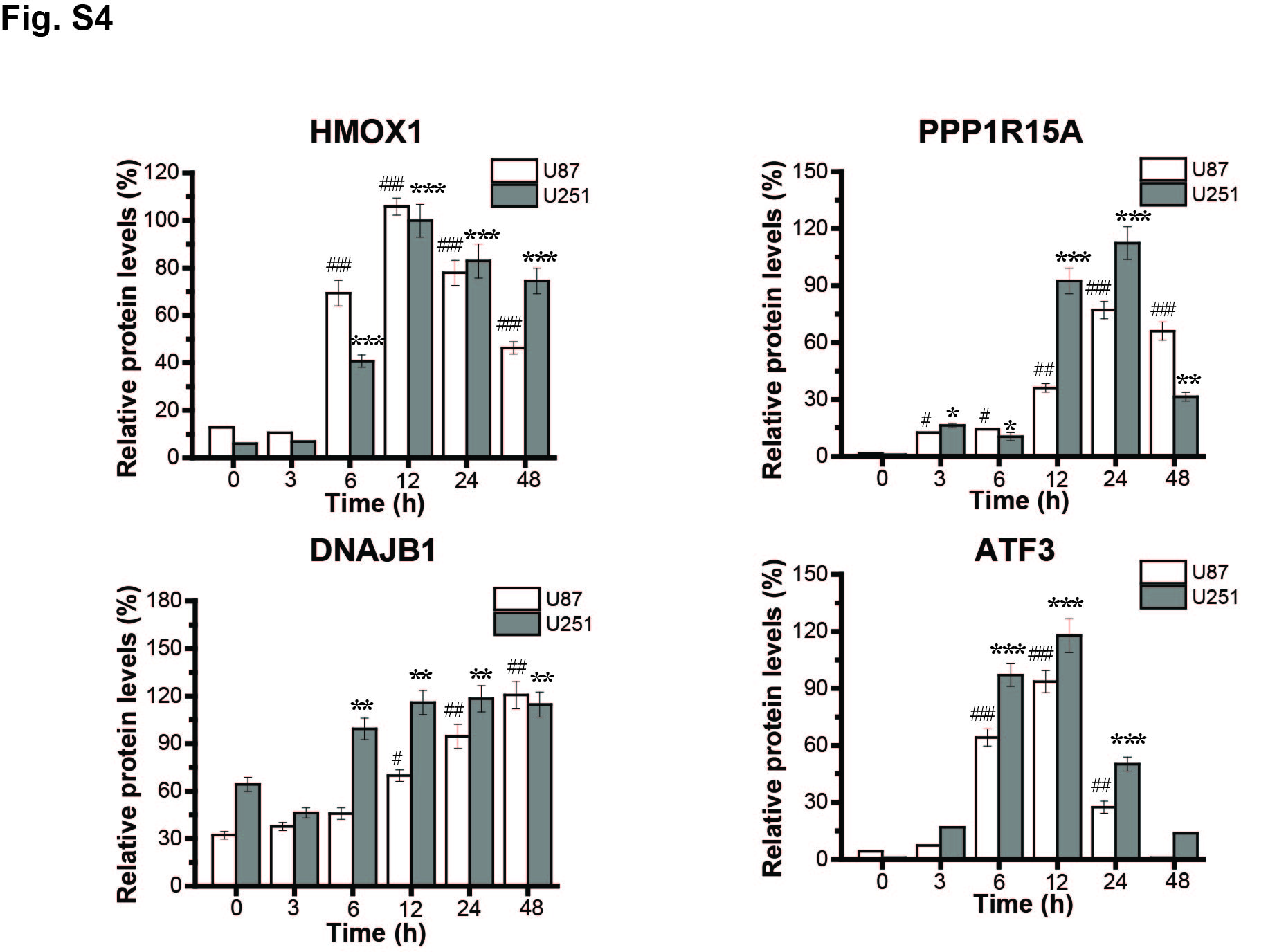

Supplement: Supplementary file 4 [file CPR-53-e12706-s004.jpg]

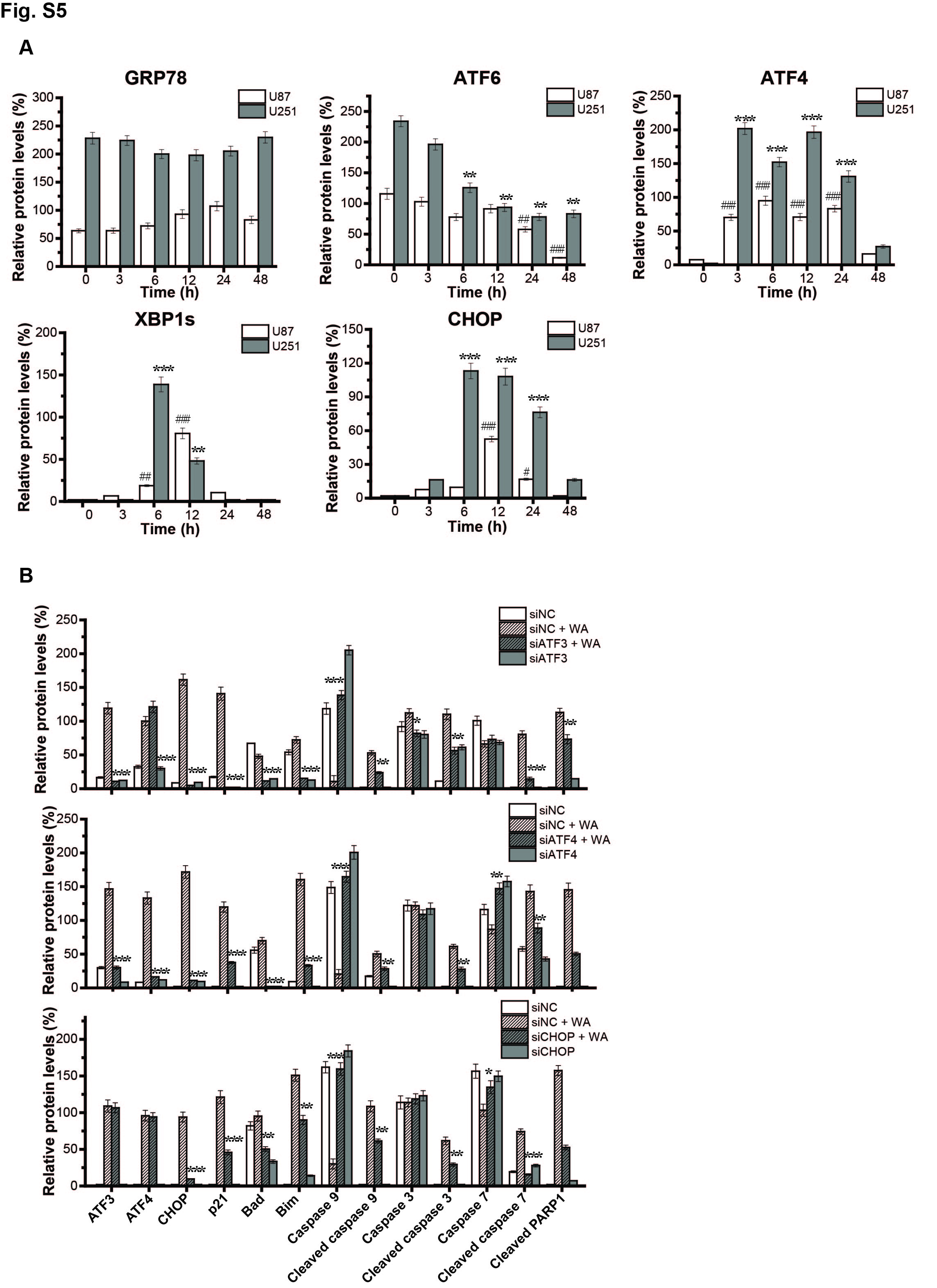

Supplement: Supplementary file 5 [file CPR-53-e12706-s005.jpg]
