# Supplementary material for: Withaferin A triggers G2/M arrest and intrinsic apoptosis in glioblastoma cells via ATF4‐ATF3‐CHOP axis
Source: Cell Prolif. 2019 Oct 23;53(1):e12706. doi: 10.1111/cpr.12706 (PMC6985693; doi:10.1111/cpr.12706)
Supplement: Supplementary file 6 [file CPR-53-e12706-s006.doc]

**Table**

**Table S1** The ID code and dilution of antibodies

| Antibodies | Company | ID code | Dilution |
| --- | --- | --- | --- |
| Cleaved PARP1 | Cell Signaling Technology | 5625 | 1:1000 |
| Caspase 3 | Cell Signaling Technology | 9662 | 1:500 |
| Cleaved caspase 3 | Cell Signaling Technology | 9664 | 1:500 |
| Caspase 7 | Cell Signaling Technology | 12827 | 1:500 |
| Cleaved caspase 7 | Cell Signaling Technology | 8438 | 1:500 |
| Caspase 9 | Cell Signaling Technology | 9508 | 1:500 |
| Cleaved caspase 9 | Cell Signaling Technology | 20750 | 1:500 |
| p-Ser (112) Bad | Cell Signaling Technology | 5284 | 1:500 |
| Bad | Cell Signaling Technology | 9239 | 1:500 |
| Bim | Cell Signaling Technology | 2933 | 1:500 |
| Bax | Proteintech | 50599-2-Ig | 1:1000 |
| Bak | Cell Signaling Technology | 12105 | 1:500 |
| Bcl-2 | Proteintech | 12789-1-AP | 1:1000 |
| Bcl-xL | Cell Signaling Technology | 2764 | 1:1000 |
| p53 | Proteintech | 10442-1-AP | 1:1000 |
| p21 | Cell Signaling Technology | 2946 | 1:500 |
| p-Thr (161) CDK1 | Cell Signaling Technology | 9114 | 1:500 |
| CDK1 | Cell Signaling Technology | 9116 | 1:500 |
| Cyclin A | Proteintech | 18202-1-AP | 1:500 |
| Cyclin B | Proteintech | 55004-1-AP | 1:500 |
| HMOX1 | Proteintech | 10701-1-AP | 1:500 |
| PPP1R15A | Proteintech | 10449-1-AP | 1:500 |
| DNAJB1 | Proteintech | 13174-1-AP | 1:500 |
| ATF3 | Cell Signaling Technology | 18665 | 1:500 |
| GRP78 | Proteintech | 11587-1-AP | 1:500 |
| ATF6 | Proteintech | 24169-1-AP | 1:500 |
| ATF4 | Proteintech | 10835-1-AP | 1:500 |
| XBP1s | Cell Signaling Technology | 40435 | 1:500 |
| CHOP | Proteintech | 15204-1-AP | 1:500 |
| GAPDH | Cell Signaling Technology | 5174 | 1:2000 |

**Table S2** The primers used in RT-qPCR

| Primers | Sence | Anti-sense |
| --- | --- | --- |
| HMOX1 | 5'-TTTGAGGAGTTGCAGGAGC-3' | 5'-AGGACCCATCGGGAAGC-3' |
| DNAJB1 | 5'-GATGGCTCTGATGTCATTTATC-3' | 5'-GCCTTCTCAGGAACTTTT-3' |
| PPP1R15A | 5'-AACCTCTACTTCTGCCTTGTC-3' | 5'-CTGGCTCCTTTACTTCTTTCT-3' |
| ATF3 | 5'-CAAAGTGCCGAAACAAGA-3' | 5'-CTGAGCCCGGACAATACA-3' |
| SH3BGR | 5'-ATCTTCTGGGTCCATAGC-3' | 5'-TCCACCTCCTGTTGTCTT-3' |
| GRP78 | 5'-TCCTATGTCGCCTTCACT-3' | 5'-ACAGACGGGTCATTCCAC-3' |
| ATF6 | 5'-AGGGTTAGAGGCGAGATT-3' | 5'-TCATAGGTCCATAGTTCAGTA-3' |
| ATF4 | 5'-GGTTCTCCAGCGACAAGG-3' | 5'-CCCAACAGGGCATCCAAG-3' |
| XBP1 | 5'-GGATTCTGGCGGTATTGA-3' | 5' AAAGGGAGGCTGGTAAGG-3' |
| CHOP | 5'-ACCAGGAAACGGAAACAG-3' | 5'-TCACCATTCGGTCAATCA-3' |
| GAPDH | 5'-AAGAAGGTGGTGAAGCAGG-3' | 5'-TTGACAAAGTGGTCGTTGAG-3' |

**Table S3** The siRNA duplexes used in this study

| Genes | Sence | Anti-sense |
| --- | --- | --- |
| Negative control | 5'-UUCUCCGAACGUGUCACGUTT-3' | 5'-ACGUGACACGUUCGGAGAATT-3' |
| ATF3 | 5'-UAUCUGUUGGAUAAAGAGGUU-3' | 5'-CCUCUUUAUCCAACAGAUAAA-3' |
| p21 | 5'-GCAUGACAGAUUUCUACCATT-3' | 5'-UGGUAGAAAUCUGUCAUGCTT-3' |
| Bim | 5'-CCCAUGAGUUGUGACAAAUTT-3' | 5'-AUUUGUCACAACUCAUGGGTT-3' |
| Bad | 5'-GGAUGAGUGACGAGUUUGUTT-3' | 5'-ACAAACUCGUCACUCAUCCTT-3' |
| ATF4 | 5'-CCCUUCAGAUAAUGAUAGUTT-3' | 5'-ACUAUCAUUAUCUGAAGGGTT-3' |
| CHOP | 5'-GCUAGCUGAAGAGAAUGAATT-3' | 5'-UUCAUUCUCUUCAGCUAGCTT-3' |
| XBP1 | 5'-GGUAUUGACUCUUCAGAUUCA-3' | 5'-AAUCUGAAGAGUCAAUACCGC-3' |
| DNAJB1 | 5'- CAUUCGAAACGAAGACAAAAU-3' | 5'-UUUGUCUUCGUUUCGAAUGCU-3' |
| HMOX1 | 5'-CAACAAAGUGCAAGAUUCUGC-3' | 5'-AGAAUCUUGCACUUUGUUGCU-3' |

**FIGURE LEGENDS**

**FIGURE S1** **WA induced the apoptosis of U87 and U251 cells via intrinsic pathway**. **A**. Quantification of apoptotic proteins after 3 μM WA treatment for various time in U87 and U251 cells. **P<0.01 and ***P<0.001 indicated the significant differences between WA-treated group and control group in U251 cells. ^##^ P<0.01 and ^###^P<0.001 represented the significant differences between WA-treated group and control group in U87 cells. **B**. Quantification of apoptotic proteins in U251 cells after 3μM WA treatment with or without Emiricasan (50 μM) pretreatment. ***P<0.001 represented the significant differences between Emiricasan-treated group and corresponding non-Emiricasan-treated group.

**FIGURE 2** **WA induced the apoptosis of U87 and U251 cells partly by up-regulating expression of Bim and Bad**. **A**. Quantification of apoptotic regulators after 3 μM WA treatment for various time in U87 and U251 cells. **P<0.01 and ***P<0.001 indicated the significant differences between WA-treated group and control group in U251 cells. ^#^P<0.05, ^##^ P<0.01 and ^###^P<0.001 represented the significant differences between WA-treated group and control group in U87 cells. **B**. Quantification of apoptotic regulators in U251 cells after 3 μM WA treatment with or without Bad or Bim siRNA duplexes pre-transfection. **P<0.01 and ***P<0.001 represented the significant differences between target siRNA-treated group and corresponding siNC-treated group.

**FIGURE 3** **Cell cycle was arrested at the G2/M phase by WA through p53-independent p21 up-regulation**. **A**. Quantification of cell cycle associated regulators after 3μM WA treatment for various time in U87 and U251 cells. *P<0.05, **P<0.01 and ***P<0.001 indicated the significant differences between WA-treated group and control group in U251 cells. ^#^P<0.05, ^##^ P<0.01 and ^###^P<0.001 represented the significant differences between WA-treated group and control group in U87 cells. **B**. Quantification of cell cycle associated regulators in U251 cells after 3μM WA treatment with or without p21 siRNA duplexes pretransfection. ***P<0.001 represented the significant differences between target siRNA-treated group and corresponding siNC-treated group.

**FIGURE 4** **Expression of HMOX1, DNJB1 and ATF3 was induced by WA at early stage of treatment**. Quantification of HMOX1, PPP1R15A, DNAJB1 and ATF3 after 3μM WA treatment for various time in U87 and U251 cells. *P<0.05, **P<0.01 and ***P<0.001 indicated the significant differences between WA-treated group and control group in U251 cells. ^#^P<0.05, ^##^ P<0.01 and ^###^P<0.001 represented the significant differences between WA-treated group and control group in U87 cells.

**FIGURE 5** **WA induced the apoptosis and G2/M arrest of GBM cells by ATF4-ATF3-CHOP axis. A**. Quantification of ER stress associated regulators after 3μM WA treatment for various time in U87 and U251 cells. **P<0.01 and ***P<0.001 indicated the significant differences between WA-treated group and control group in U251 cells. ^#^P<0.05, ^##^ P<0.01 and ^###^P<0.001 represented the significant differences between WA-treated group and control group in U87 cells. **B**. Quantification of apoptosis and cell cycle associated regulators in U251 cells after 3μM WA treatment with or without ATF3, ATF4 and CHOP siRNA duplexes pretransfection. *P<0.05, **P<0.01 and ***P<0.001 represented the significant differences between target siRNA-treated group and corresponding siNC-treated group.
